# The Effects of Ozone

**Published:** 1882-02

**Authors:** 


### THE EFFECTS OF OZONE.

Dr. Barlow, in a recent work upon the effects of ozone on the animal economy, has not arrived at the same conclusions as Ireland, Richardson, Redfern, and others. His conclusions are as follows :—

1. Ozone depresses the nervous system, an effect probably secondary, and due to the excess of carbonic acid in the blood.

2. It lowers the normal number of respirations, and diminishes the force and frequency of the heart's action.

3. Inhalations of ozonized air diminishes the normal quantity of carbonic acid eliminated and of oxygen absorbed.

4. The diminution of the number of respirations which retards the movements of the heart, and the diminution of the quantity of carbonic acid exhaled, are probably due to the action of the ozone upon the pulmonary mucous membrane.

5. The alteration of the mucus may, if the ozone inspired be in great quantity, or if the air expired contain enough carbonic acid, induce death in an hour, from asphyxia

6. If the quantity of the ozone is small, the alteration in the mucous membrane does not kill by asphyxia, but death is due to bronchitis.

7. Death by bronchitis can occur after a sojourn of an hour in an atmosphere containing one per cent of ozone.

8. Ozonized air in direct contact with the blood decolorizes the red corpuscles, arrests the amœboid movements of the leucocytes, renders visible the nuclei of the globules, and gives life to the granulations in the serum.

9. The change of color of the globules is due probably to the combination of the ozone with the hæmoglobin.

CURE FOR HICCUP. "Frighten me. What for?" "I've the hiccups. Frighten me and they'll go away." "All right. Lend me five francs?" "Thanks, they're gone."

